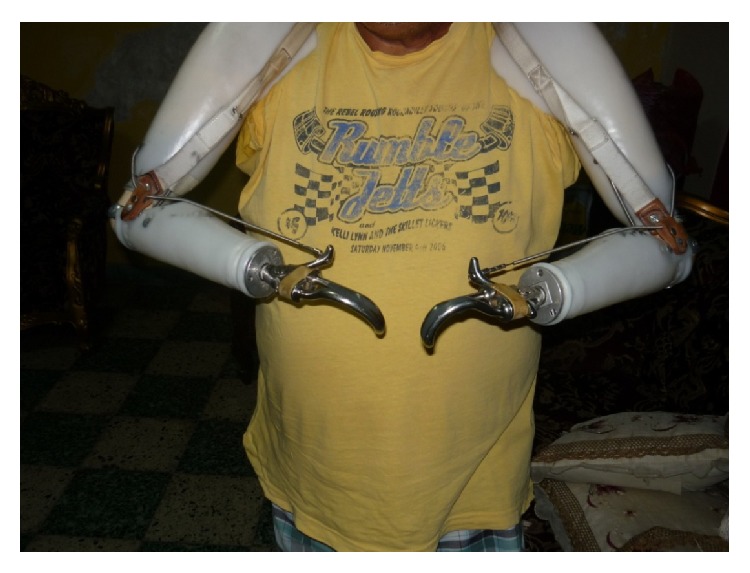# Severe Burns and Amputation of Both Arms in the First Psychotic Episode of a Schizophrenic Patient

**DOI:** 10.1155/2015/405713

**Published:** 2015-08-31

**Authors:** Lizardo Cruzado, Ronald Villafane-Alva, Katia Caballero-Atencio, Carla Cortez-Vergara, Patricia Núñez-Moscoso

**Affiliations:** ^1^Instituto Nacional de Salud Mental “Honorio Delgado-Hideyo Noguchi”, Jr. Eloy Espinoza 709, Urbanización Palao, San Martín de Porres, Lima 31, Peru; ^2^Facultad de Medicina Alberto Hurtado, Universidad Peruana Cayetano Heredia, Avenida Honorio Delgado 430, Urbanización Ingeniería, San Martín de Porres, Lima 31, Peru; ^3^Facultad de Medicina de San Fernando, Universidad Nacional Mayor de San Marcos, Avenida Grau 755, Lima 1, Peru

## Abstract

An alleged reduction of sensitivity to pain in people with schizophrenia has been reported, but the nature of this complex phenomenon has not been elucidated yet. Reports of insensitivity to burns from people with schizophrenia are extremely rare. We report the case of a 24-year-old man who set both of his arms on fire during the first break of paranoid schizophrenia. As a result of severe tissue damage, both of his limbs had to be amputated. Today, at the age of 59, the patient is physically and mentally rehabilitated and is adherent to treatment. Additionally, given the uncertainty about the true nature of the alleged hypoalgesia in schizophrenia, we postulate the need for a comprehensive phenomenological approach in the study of embodiment in people with this condition.

## 1. Introduction

Five to 13% of people with schizophrenia will commit suicide and often do so by unusual methods, including incineration [1, 2], and especially in the early stages of the disease. On the other hand, approximately 11% of patients with schizophrenia injure themselves at some point between the beginning of their psychosis and their first psychiatric consultation [3]. It is striking, however, that the case reports on self-injury due to burns in schizophrenic patients are scarce [4], although it is known that a significant percentage of self-inflicted burns are performed by people with severe mental illnesses, including schizophrenia [5, 6].

Although the relative insensitivity to pain in people with schizophrenia had already been discussed since the classic texts of Kraepelin and Bleuler, the mechanisms responsible for this peculiarity have not been fully clarified so far. Some authors claim a real hypoalgesia among patients with schizophrenia while others simply declare a decline in the observable behavioral response to pain [7, 8]. The evidence that supports an apparent hypoalgesia in patients with schizophrenia contains (1) case reports in which conditions such as peritonitis, femur fracture, or myocardial infarction provoked a minimal expression of pain, (2) population studies where either lower prevalence of painful conditions in people with schizophrenia, or the opposite, lower prevalence of people with schizophrenia among the population suffering from chronic painful conditions, were detected, and (3) experimental studies in which a different pain threshold was recorded in people with schizophrenia [9].

Criticism of these studies include the small sample size, the simulating analgesic effect of antipsychotic drugs, the lack of distinction between pain perception and its behavioral expression, the lack of homogeneity among the samples studied (schizophrenia is a very multifaceted pathology), and the use of multiple experimental approaches, as well as the lack of discernment among sensory, emotional, and behavioral components involved in the painful experience of the individuals studied [7, 8]. Just the same, clinical studies that report hypoalgesia in schizophrenia should be compared with the experimental studies in which increased sensitivity to pain was found [10–12]. All these discrepancies can be summarized in the difficulty to objectify pain, which is a highly subjective phenomenon.

In order to discuss briefly the current state of research about pain in people with schizophrenia, we present the case of a young man who, during the first outbreak of his schizophrenia, intentionally provoked severe burns on both of his arms, which had to be amputated.

## 2. Case Presentation

We present the case of a 59-year-old single male with incomplete higher technical education and a maternal history of schizophrenia. The onset of his symptoms came at age 24 (in 1980) when he realized that a couple of his colleagues in the shops where he used to work started to plot against him in the course of a few days. Full of anxiety, he left the place quickly but when getting on a taxi, he seemed to notice that the taxi driver was also part of the conspiracy. When he got home he felt also threatened: he feared that his enemies were to invade his home, so he ran away; in the confusion of his mind he thought to flee to the United States. On his journey, he walked eight kilometers orienting himself “by the flight of birds” until he reached the sea. On his way, he experienced several delusional perceptions and once he reached the beach, he plunged into the water following a light he had glimpsed on the horizon, convinced “that God was leading him.” After several hours of praying, the waves finally dragged him back to the shore.

He was taken to his home by the police. There his relatives perceived him to be perplexed and evasive. The patient remembered everything that happened and, despite his long journey, he did not feel tired. He refused food because he believed fasting was essential in his “training.” He was still convinced of being followed, and he was also convinced of perceiving the threatening stares of his neighbors through his home windows. Then he came up with a subterfuge: he would make his enemies believe that he had killed himself so that they would give up their hostilities.

In order to do that, he went to his bedroom, carrying newspapers and kerosene. He mounted a pyre and burned it down. His idea was to convince his neighbors that he had committed suicide by burning himself but, at once, he assumed that this should be part of a bizarre ritual of “consecration.” While he recited the Lord's Prayer, he approached the fire with his eyes closed and his clothes ignited. Our patient just remembers having experienced a slight feeling of heat but no pain. His relatives found him praying aloud, with his arms still on the pyre and, surprisingly, the patient looked indifferent. “What really was preoccupying me, when I was removed from the fire, is that I hadn't finished my ritual yet”; he explained later, “At every moment when this was happening, I felt like an automaton.”

He recalls that, on the way to hospital, he saw the street lights as torches that gave off toward his “mission” and that only at the fifth day he fully experienced the pain of his injuries. Due to the seriousness of his burns, which included his arms, neck, and chest, successive amputation of his upper limbs was performed within the first six months. During his hospitalization the patient began antipsychotic treatment with 800 mg of thioridazine. This caused him intolerable drowsiness; therefore treatment was changed to 40 mg of trifluoperazine, with progressive improvement of psychotic symptoms. This psychotic episode lasted for about four months. After that, the patient was discharged. The patient remembers that, in that time, he still believed that skin grafts were part of hospital's scientific experiment and everything was part of a typical “ritual of preparing” of the Japanese religion which was professed by the owner of the shop where he had worked.

The patient remained stable and relatively symptom-free on trifluoperazine. It was not easy to assess his level of functionality because of his significant physical disability. A year later, he felt himself depressed by his situation and developed suicidal ruminations but he recovered without antidepressant drugs, spontaneously, after some months. He remained housebound watching TV or reading newspapers; he had limited communication with others and no social life.

Twelve years after the first break, at the age of 37, the patient was persuaded by relatives to stop treatment, as they felt he was not ill. A week after treatment was interrupted, he experienced global insomnia, auditory hallucinations, and paranoid delusions. He perceived secret messages on radio and television shows. He was convinced of possessing telepathic powers and attacked his mother. Therefore, antipsychotic treatment was restarted on fluphenazine decanoate 25 mg biweekly.

In the following years, although he continued saying that he could see the future and that he had telepathic powers, he remained relatively stable. When he interrupted treatment because of parkinsonian side effects, soliloquies, insomnia, psychomotor restlessness, hostility, and catatonic behavior quickly reappeared. As such, oral antipsychotics were preferred over long acting injectable antipsychotics. When trying to restart his technical studies, the patient found it impossible since he felt tired easily and his concentration had become poor.

**Figure 1:**
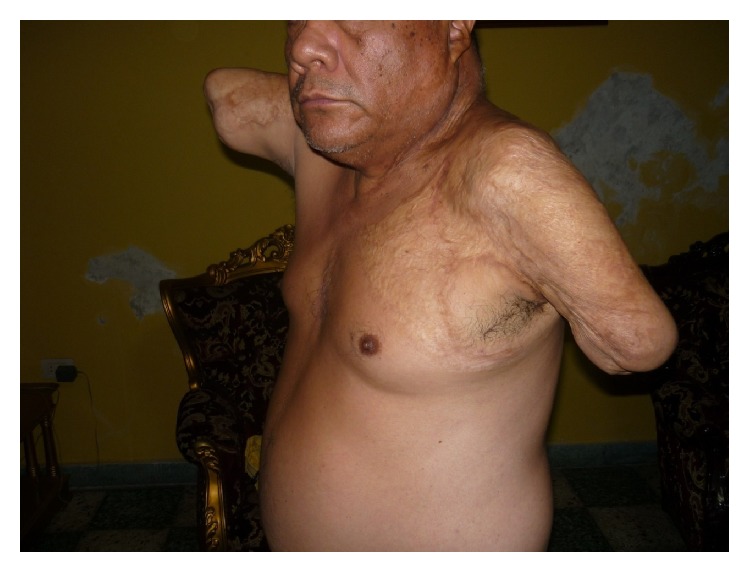


He suffered a relapse in symptoms following a strong earthquake eight years ago. His paranoid, mystical, and thought withdrawal delusions reappeared as well as his aggressiveness towards his family, so he was hospitalized for three months. Currently he is under treatment with fluphenazine decanoate and risperidone. Although sometimes he feels sad for everything that happened, this does not affect his lifestyle, and usually he feels in good mood. He lives with his mother and works as a pastor of an evangelical church. He is also fond of reading novels and newspapers. The patient uses traction powered functional mechanical prostheses with hooks as upper limbs (Figures 1 and 2). With these devices he can dress himself, feed himself, and use simple tools.

## 3. Discussion

Pain is a multidimensional phenomenon which includes sensory (intensity, nature, duration, and location of pain), emotional (galling characteristics of pain), and cognitive (mental processes that give meaning to pain perception and articulate behavioral response) components, which implies that pain is often an individual and subjective experience [7, 8]. However, the archaic paradigm that characterized the psychiatric patient as insensitive to the rigors of nature persisted until the eighteenth century, and it has subsequently influenced the description of the alleged analgesia of people with schizophrenia. It should be noted that different experiences and reactions to pain are also seen in other neuropsychiatric disorders such as major depression and bipolar disorder, eating disorders, Alzheimer's dementia, Parkinson's disease, and some personality disorders [13]. However, the nature of these phenomena has not been clarified and the neurobiological findings are equally of mixed nature. For example, in people with depression, hyperalgesia has been found secondary to ischemic stimuli; however, hypoalgesia has been found with the application of thermal stimuli [14].

The latest bibliographical evidence incites the discussion about pain sensitivity in schizophrenia. Certainly, restricted assessment to case reports or experimental studies does not fully represent the knowledge of pain experience in schizophrenia. For example, Stubbs et al. [15], in a population-based study, found prevalence of clinical pain pathologies in 34.7% of people with schizophrenia but also in a similar proportion of people without it. Furthermore, in a meta-analysis, Engels et al. [10] found that even when people with schizophrenia present a lower expression of pain in specific medical conditions such as myocardial infarction or post lumbar puncture, they do not make such distinction in the experience of pain without a specific cause. Apparently, a crucial aspect regarding this topic is the difference between experimental pain and clinical pain, so that the first cannot be easily compared to the actual experience of the latter. The difference between these paradigms has been reported previously, subsisting in their genesis imbalance between spinothalamic tract areas responsible for processing the clinical pain (with serotonergic and adrenergic neurotransmitters) and experimental pain (opioid neurotransmitters) [7]. A recent experimental study that may reconcile these two perspectives is that of Girard et al. [16], where hyperalgesia was found due to acute and limited stimuli but where hypoalgesia was reached with the repetition of such stimuli. This may be explained by lack of the mechanism for pain sensitization in people with schizophrenia.

Other discrepancies result from the association of schizophrenic hypoalgesia with different symptom clusters of this psychosis, for example, negative symptoms such as apathy, anhedonia, and affective flattening, especially in advanced stages of the disease. In this way, the indifference to the affective component of pain correlates with the emotional restriction of people with schizophrenia [17]. On the other hand, the positive symptoms of the disease have been associated with both increase and decrease of sensitivity to pain, depending on the types of studies performed [7]. Furthermore, it has been described that the cognitive impairment of schizophrenia might justify the abnormal expression of the painful experience because of the attention deficits of patients, the difficulty of accessing their mnemonic catalog of prior pain responses, and reduced processing speed, as well as the fact that people with schizophrenia lack spontaneity and responsiveness to show their pain complaints, causing a false impression of hypoalgesia in the examiners [8]. In general, the heterogenous clinical manifestations of schizophrenia may explain phenomena classified as hypoalgesia in acutely psychotic patients that go as far as removing one of their eyes in order to obey biblical injunctions deliriously interpreted, as well as in chronically impaired and autistic subjects, alienated and minimally bewildered in the face of their own pain experience [7, 8]. It is obvious that these phenomena do not necessarily share the same neurobiological basis and should not be explained by the same research paradigm [18].

In short, the current scientific view considers that, rather than being an alleged hypoalgesia in schizophrenia, this phenomenon is a different way of experiencing and expressing pain [7, 8]. This is also supported and is consistent with a wide range of global changes in the experience of corporeality and relationships of the schizophrenic self with the surrounding world, perturbations which are increasingly receiving attention (e.g., cenesthetic disturbances, passivity experiences, and body image and body schema distortions, to mention the most prominent). So to speak, the schizophrenic patient does not “live” in his body anymore, since he does not use it as a means to interact with the inner and outer world [19]. Thus, the alteration of the pain experience would become one of the phenomena emerging from here [20].

These phenomena have been ordered in the concept of “disembodiment of the self,” which, at the same time, is one of the first symptoms of schizophrenia and deep core of ipseity disturbances (ipse is Latin for self or itself, and it is synonymous of “minimal self” or “core self,” that is to say, the basic sensorial nucleus in which the “self” is laid down) [21]. It also provides an interpretative framework for self-mutilation phenomena and extreme indifference to pain as in the case we reported [22]. Copious recent scientific papers point to the consideration of these phenomena as the basis of the nature of the schizophrenic experience [23, 24]. Even, it has been pointed out that the essential feature of schizophrenic existence is its being disembodied [19, 20]. Even feelings of not belonging to one's body and severe body schema disturbances are verified as being still present in the patients since their first psychotic episodes [25], which is probably what happened in the case we have reported. Given the elapsed time since the first psychotic episode of our patient, it was not possible to explore in detail his experiences of ipseity and embodiment; nevertheless, in the patient's narration we can detect experiences of disembodiment (the distance he felt between his subjectivity and his body while his arms were burning, to the point he felt his own arms as objects), and there were also apparent alterations in his sense of agency, as the patient felt, in his own words, “like an automaton.” [22, 23].

The fact that positive symptoms are correlated with both hypo- and hyperalgesia in acute episodes of schizophrenia suggests that the characteristics of the cases associated with either finding have not been individualized [7]. Therefore, in the context of pain experience phenomenological scrutiny of acute-onset schizophrenia, with its presence of ecstasy, mysticism, and other disturbances of consciousness [26, 27], is pending. So far, phenomenological assessment of corporeality in schizophrenic patients, in relation to their different pain experiences, has not been conducted, even when it is a perspective that could contribute to the complete understanding of this phenomenon [28].

In the presence of a phenomenon of extreme complexity as the experience of pain, we should consider Nagel's aphorism: “A necessary requirement for any coherent reductionism is that the entity to be reduced is properly understood” [29]. Therefore, apart from the experimental and epidemiological findings on pain mechanisms, the full understanding of this phenomenon requires the essential consideration of the phenomenological perspective to approach as closely the first-person experience of those affected [21, 30].

The case of our patient is not limited to the barbaric mutilation that he suffered at the beginning of his psychosis, but it is a valuable example of how, despite his physical and mental limitations, this person has managed to overcome them and made a decent living, contradicting Wittgenstein's dictum: “The human body is the best picture of the human spirit.” Here the opposite is true: the body of a human being suffering from schizophrenia, with all the changes of its most intimate bodily experience, and even in the extreme circumstances of his mutilations, can go and arrive further.

## Figures and Tables

**Figure 2:**